# Somatic and germline analyses of a long term melanoma survivor with a recurrent brain metastasis

**DOI:** 10.1186/s12885-015-1927-0

**Published:** 2015-11-23

**Authors:** Sarah Weiss, Farbod Darvishian, Jyothi Tadepalli, Richard Shapiro, John Golfinos, Anna Pavlick, David Polsky, Tomas Kirchhoff, Iman Osman

**Affiliations:** Deparment of Medicine, New York University School of Medicine, 522 First Ave., Smilow Building Room 403, New York, NY 10016 USA; Interdisciplinary Melanoma Cooperative Group, New York University School of Medicine, 522 First Ave., Smilow 403, New York, NY 10016 USA; Department of Pathology, New York University School of Medicine, 540-562 First Ave., New York, NY 10016 USA; The Ronald O. Perelman Department of Dermatology, New York University School of Medicine, 522 First Ave., Smilow 4th floor, New York, NY 10016 USA; Department of Surgery, New York University School of Medicine, 160 E. 34th St., 4th floor, New York, NY 10016 USA; Department of Neurosurgery, New York University School of Medicine, 530 First Ave., 8th floor, New York, NY 10016 USA; Departments of Population Health and Environmental Medicine, New York University School of Medicine, 522 First Ave., Smilow 12th floor, New York, NY 10016 USA

**Keywords:** melanoma, brain metastasis, germline, somatic, immune system

## Abstract

**Background:**

Median overall survival (OS) of patients with melanoma brain metastases (MBM) is usually 6 months or less. There are rare reports of patients with treated MBM who survived for years. These outlier cases represent valuable opportunities to study the somatic and germline factors that may have influenced patient outcome and led to extended survival.

**Case presentation:**

Here we report the clinical scenario of a 67 year old man with a recurrent brain metastasis from melanoma who has survived over 12 years post-resection. We review the literature relating to clinical and molecular variables associated with long term survival post-brain metastasis. We present the somatic characteristics of this individual patient’s tumor as well as an analysis of inherited genetic variants related to immune function.

The patient’s resected brain tumor is *BRAF* V600E mutated, *NRAS* wild type (WT), and *TERT* C250T mutated. The patient is a carrier of germline variants in immunomodulatory loci associated with prolonged survival.

**Conclusions:**

Our data suggest that genetic variants in immunomodulatory loci may partially contribute to this patient’s unusually favorable outcome and should not be overlooked. With further and future investigation, knowledge of inherited single nucleotide polymorphisms (SNPs) may provide clinicians with more individualized prognostic information for melanoma patients, with potential implications for surveillance strategies and therapeutic interventions.

## Background

Several published case reports document extended survival (defined as > 3 years) in melanoma brain metastasis (MBM) patients who had multiple brain metastases and/or concurrent extracranial disease [1, 2], however only two published case reports document extended survival of patients treated for a brain-only metastasis [3, 4]. There is no literature to our knowledge reporting on the molecular analysis of a long term survivor’s MBM.

We describe the case of a patient who was enrolled in the New York University (NYU) Interdisciplinary Melanoma Program biorepository database at the time of primary diagnosis and was actively followed up by protocol driven, institutional review board (IRB) approved guidelines who developed a single MBM and did not respond to initial gamma knife therapy. He subsequently underwent surgical resection and remained disease-free for 6 months before the brain metastasis recurred and required re-resection. As a result of the availability of tissue from his primary melanoma, the metastases to the brain, and blood samples, we had the opportunity to perform molecular and genetic analyses to investigate tumor- and host-based characteristics that might explain the unexpectedly prolonged survival of this patient.

## Materials and methods

### DNA isolation and mutation profiling

DNA was isolated from formalin-fixed paraffin embedded (FFPE) tumor sections of the resected brain metastasis using the BiOstic FFPE tissue deoxyribonucleic acid (DNA) isolation kit (MO BIO, Carlsbad, CA). The mulitplex SNaPshot assay was used to detect mutations in v-Raf murine sarcoma viral oncogene homolog B (*BRAF*) (V600), neuroblastoma RAS viral (v-Ras) oncogene homolog (*NRAS*) (Q61), and the telomerase reverse transcriptase (*TERT*) promoter (C228T (−124C > T) and C250T (−146C > T)).

### Germline analysis

The patient was included in a recent analysis testing the association of 72 single nucleotide polymorphisms (SNPs) tagging 33 interleukin genes and 11 cytokine regulatory genes (using Haploview Tagger) with melanoma survival in 1022 cutaneous melanoma patients [5, 6]. SNP genotyping was performed using the MassARRAY iPLEX platform. Associations between immunomodulatory SNPs and recurrence-free survival (RFS) and overall survival (OS) was tested using Cox models.

## Case presentation

**Fig. 1:**
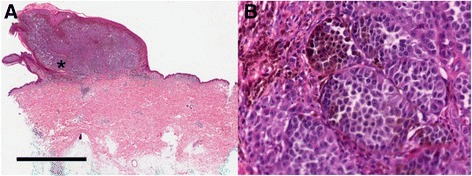
Photomicrograph of H&E stained primary nodular melanoma at (**a**) low and (**b**) high magnification showing nests of atypical melanocyte proliferationsmalignant melanoma tumor cells. The scale bar represents 4 mm for panel A and 125 μM for panel B

The patient is a 67 year old man with a history of asthma and seasonal allergies who developed a pigmented lesion on his lower back. The lesion was excised and showed a non-ulcerated, 3.92 mm thick invasive melanoma with 2 mitoses per square millimeter, non-brisk tumor-infiltrating lymphocytes (TILs), and partial regression (Fig. 1). There was no lymphovascular invasion. Wide excision showed negative margins and no residual melanoma. A left inguinal sentinel lymph node biopsy was negative, and the right inguinal sentinel lymph node contained a 0.15 mm micrometastatic focus of melanoma in the subcapsular region. There was no evidence of distant disease at diagnosis and AJCC staging was determined to be stage IIIA (T3aN1aM0).

The patient received one year of adjuvant high dose interferon-alfa (IFNα) with 20 million units/m^2^ as intravenous induction therapy followed by maintenance therapy with 10 million units/m^2^ subcutaneously three times per week.

**Fig. 2:**
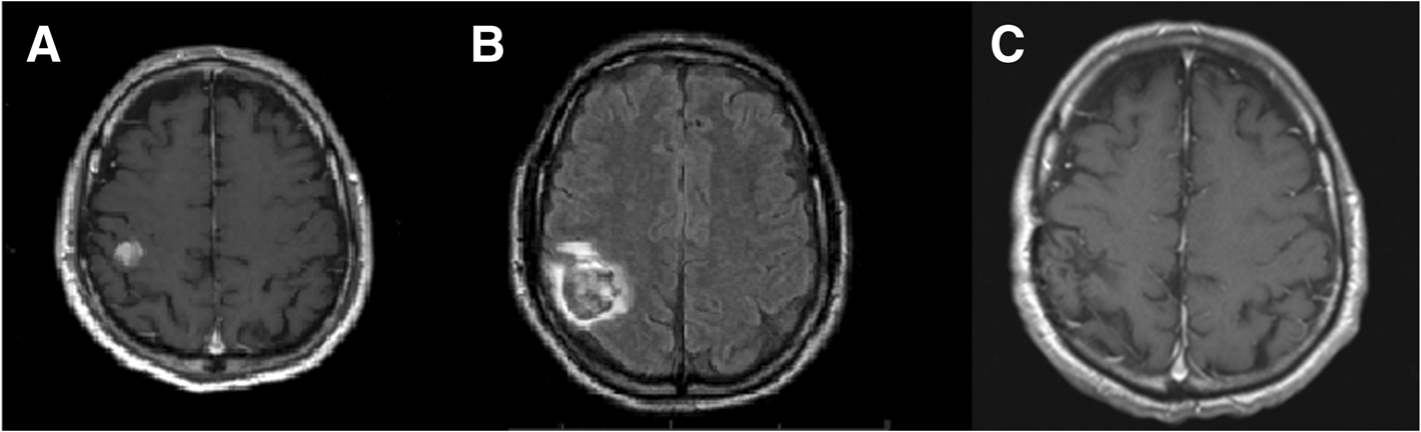
Coronal contrast-enhanced MRI brain images showing (**a**) initial 1.3 cm right parietal lobe metastasis without mass effect or cerebral edema; (**b**) enlarging 4 cm hemorrhagic right parietal mass with vasogenic edema 6 weeks post-gamma knife; (**c**) no evidence of brain metastases 12 years after resection

**Fig. 3:**
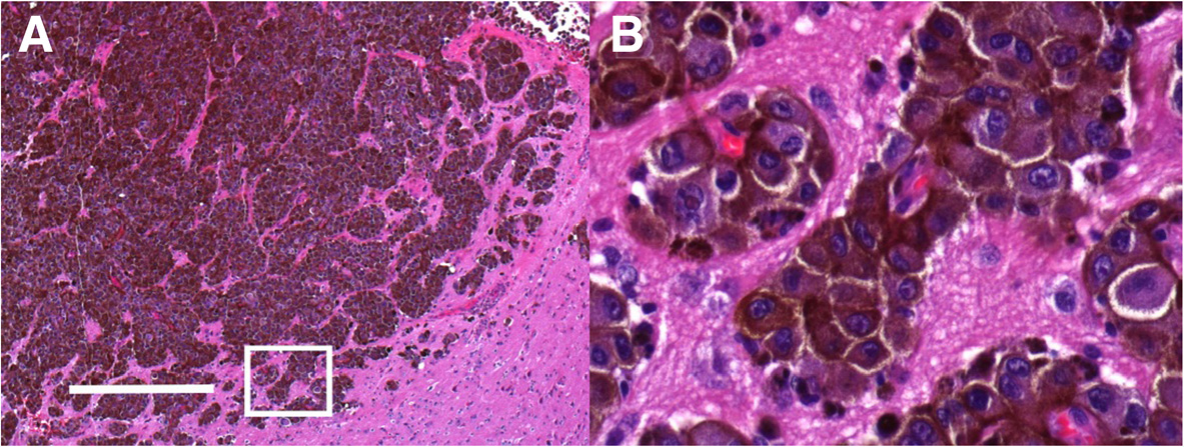
Photomicrographs of H & E stained right parietal lobe mass excision at (**a**) low and (**b**) high magnification. The scale bar represents 400 μM for panel a and 50 μM for panel b. on (A) low and (B) high magnification demonstrating metastatic melanoma with non-brisk TILs and areas of mild to moderate hemorrhage

The patient underwent routine clinical exams and surveillance imaging at pre-specified intervals. A year and a half after completing adjuvant IFNα, surveillance imaging with a brain computed tomography (CT) scan showed a 1.3 cm brain metastasis in the right parietal lobe without mass effect or cerebral edema (Fig. 2a). He was referred to a neurosurgeon at our institution and underwent gamma knife of the single brain metastasis. Six weeks post-gamma knife, follow-up brain magnetic resonance imaging (MRI) demonstrated an enlarging hemorrhagic right parietal mass measuring 4 cm with vasogenic edema (Fig. 2b). The patient underwent right parietal craniotomy, with the pathology revealing metastatic melanoma (Fig. 3) positive for S100, human melanoma black 45 (HMB-45), and variable mindbomb E3 ubiquitin protein ligase 1 (MIB1) staining. Post-operative MRI imaging documented total resection. He recovered well from surgery, was maintained on levetiracetam for seizure prophylaxis, returned to work, and continued follow-up with the medical oncologist and neurosurgeon.

Six months post-craniotomy, the patient presented with focal seizure activity and was found to have a new enhancing right parietal mass measuring 1.7 × 2.1 × 1.5 cm with surrounding vasogenic edema (imaging unavailable). He underwent re-resection of the mass, and pathology was consistent with recurrent melanoma. Radiographic staging showed no evidence of extracranial metastases. He underwent a 6 month course of adjuvant therapy with temozolomide 200 mg/m^2^ daily for 5 days, every 28 days.

After completing temozolomide, the patient continued to undergo surveillance brain MRIs every 6 months which was extended to yearly intervals for a total of 10 years. He had no further evidence of melanoma recurrence and continues to have regular follow-up with his medical oncologist and his dermatologist. He remains melanoma-free over 12 years post brain metastasis resection (Fig. 2c).

**Fig. 4:**
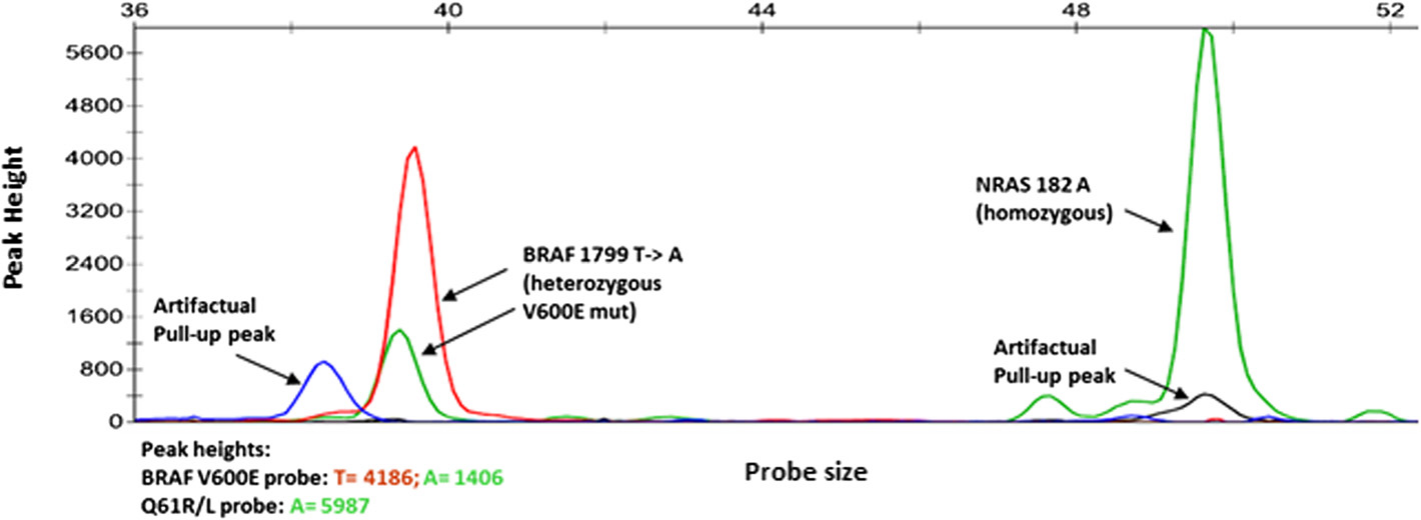
Multiplex SNaPshot assay performed on resected brain metastasis demonstrating the tumor to be BRAF V600E mutant and NRAS wild type. Color codes: green = A, red = T, black = C, blue = G

The patient’s resected brain tumor is *BRAF* V600E mutated, *NRAS* wild type (WT), and *TERT* C250T mutated (Fig. 4). The patient is a carrier of germline variants in immunomodulatory loci associated with prolonged survival. We found a significant association with melanoma OS for 2 independent genetic variants (rs3024493, r222202) in the interleukin locus at 1q32.1, mapping in the region of interleukin 10 (IL10) [5]. As noted in Table 1, for these 2 variants, the patient carries the genotypes that associate with significantly extended OS in a population-level analysis.

**Table 1:** Patient’s genotypes found to be associated with improved survival outcomes at the population level

SNP	Gene	Patient genotype	OS
			HR; *p*-value
rs3024493	IL10	GT	0.58; *p* = 0.00069
rs222202	IL10	CT	0.58; *p* = 0.0042

## Discussion

In 2014, there were over 76,000 new melanoma cases and over 9000 melanoma deaths in the United States [7]. The incidence of MBM ranges from 10 %–50 % in clinical population-based studies [8, 9] and 45 %–75 % in autopsy series [10]; however, incidence is increasing due to improved MRI accessibility and requirements for baseline brain imaging in melanoma clinical trials [11]. Multiple MBM are more common than single brain metastases [10]. Independent risk predictors for MBM in the primary tumor include location (trunk, head and neck, or mucosa) and presence of ulceration [12].

Median OS from the time of diagnosis of MBM is less than 12 months, depending on the number of metastases, the extracranial disease burden, and the administered treatment. Survival rates at 6 months, 1 year, and 5 years have been reported as low as 35 %, 20 %, and 5 %, respectively [8, 12, 13]. Post-MBM survival is 12 months for patients with a solitary MBM compared to only 4 to 6 months for patients with co-existing extracranial disease [12], which represents approximately 50–60 % of patients with MBM. Factors significantly associated with worse OS include age greater than 65, presence of extracranial disease at time of brain metastasis diagnosis, increasing number of brain metastases, frontal lobe involvement, bilateral involvement, presence of neurological symptoms, weakness, and fatigue [8, 12].

This patient initially presented with stage IIIA melanoma, which carries a high risk of future recurrence and distant metastases, particularly in the first three years after diagnosis. Five year recurrence rates at any site for stage IIIA, IIIB, and IIIC are 48 %, 71 %, and 85 %, respectively [14]. The National Comprehensive Cancer Network (NCCN) guidelines recommend that patients with initially diagnosed stage IIB-IV melanomas who were fully resected undergo clinical evaluation and physical exam every 3 to 6 months for 2 years and then every 3 to 12 months for the following 2 years, with cross sectional imaging and brain MRI to be considered yearly as a category 2B recommendation. Risk of recurrence in the brain as the first site has been reported as only 5 % for stage IIIA melanoma, although this observation is impacted by surveillance practices [14]. Most recurrences are discovered within 5 years after initial diagnosis, so surveillance imaging is not recommended beyond this time point [15].

In this patient’s case, adherence to surveillance imaging recommendations identified an asymptomatic solitary brain metastasis that was not yet clinically evident. However, negative imaging studies at a single point in time are unfortunately poor predictors of future metastatic spread, which could theoretically occur at any time. Identification of biomarkers that predict risk of future recurrence or site-specific metastasis, for example to the brain, would aid in identification of patients who may require more rigorous surveillance or who may benefit most from adjuvant therapies.

There is limited published data on the molecular alterations associated with MBM, although in general, debate in the literature exists as to whether *BRAF* and/or *NRAS* mutated melanomas are associated with worse survival outcomes compared to WT tumors [16–18]. Furthermore, the available data may be confounded by prolonged survival in MBM patients treated with *BRAF* inhibitors [19]. *TERT* promoter mutations are known to be more common in *BRAF* or *NRAS* mutated tumors compared to WT melanomas and trend towards worse OS [20], however the role of *TERT* mutations in MBM is also unknown.

Chen et al. analyzed 16 pairs of matched brain and extracranial melanoma metastases for recurrent “hotspot” mutations known to be influential in cancer. *BRAF* V600E and *NRAS* Q61K mutations were found in 7/16 (44 %) and 3/16 (19 %) patients, respectively, with 100 % concordance between the matched brain and extracranial metastases. Copy number variation (CNV) analysis of 10 MBM found large chromosomal gains (>35 %) and losses with similar frequencies found in the paired extracranial metastases, however overall there were no overlapping CNV profiles among the MBM. Interestingly, protein expression profiling of 7 pairs of matched tumors demonstrated MBM to have overexpression of proteins involved in the phosphatidylinositol 3-kinase (PI3K)/v-akt murine thymoma viral oncogene homolog (AKT) pathways including AKT_pS473, GSK3β_pS9, GSK3α/β _pS21/S9, and PRAS40_pT246 [21]. In this manner, identifying molecular features unique to MBM, which represents a significant therapeutic challenge, can reveal signaling pathways implicated in MBM pathogenesis and suggest targets for “organ-specific therapy” to the brain [21]. While it is unclear whether the *BRAF* or *TERT* mutations in this patient’s tumor had an impact on his early brain-only recurrence or his eventual prolonged survival, ongoing investigation is needed to understand the implications of MBM somatic mutational profiles.

The patient is a carrier of germline variants in immunomodulatory loci associated with prolonged survival (Table 1), proposing another suggestive explanation of his unusual outcome. Notably, the low penetrant effect of these variants clearly indicates that the patient’s favorable outcome is unlikely to be attributed solely to the effect of the tested SNPs and that other genetic or non-genetic factors will impact these associations.

IL-10 has traditionally been considered an immunosuppressive cytokine, associated with more aggressive melanoma tumor progression [5, 22]. In our recent study, rs3024493 correlated with decreased IL-10 secretion by CD4+ T-cells in additive fashion (expression dependent on the dosage of minor allele). However, the association with more favorable OS was observed only in heterozygotes that associate with mid-level secretion of IL-10 [5]. This may further support the recent hypothesis that IL-10 also exerts immunostimulatory activities, as recently demonstrated in molecular studies suggesting IL-10 stimulation of interferon-gamma [23]. It is possible that this and other immunological mechanisms impact the effect of associated genetic variants in the IL10 locus. Hence, while the two germline variants show promising capacity for personalized prediction of unusual outcomes of the studied patient, their translation into clinical practice will strongly depend on the completion of more comprehensive genetic and functional studies in the future.

## Conclusions

In conclusion, we report the somatic and germline characteristics of a patient with a treated MBM who exceeded survival expectations. We highlight how data from molecular tumor profiling and genetic variant analysis may assist in understanding a patient’s clinical course. The patient’s brain metastasis was a *BRAF* V600E mutated, *NRAS* WT, *TERT* C250T mutated tumor with evidence of germline SNPs that may be associated with improved OS. We underscore the substantial role of the immune system in regulating tumor control or progression. For example, we illustrate the potential impact of the interleukin pathway in augmenting immune activation, as the patient carries a germline variant in the interleukin locus at IL10, which may partially contribute to the patient’s favorable outcome. Of note, this patient also received temozolomide post-MBM resection. While temozolomide has been associated with intracranial responses in patients with unresected, non-irradiated MBM, its low response rates suggest that it is unlikely to entirely account for this patient’s outcome [24]. Additionally, although the brain metastasis is *BRAF* V600E mutated, this molecular profiling was performed retroactively for research purposes. At the time of the brain metastasis diagnosis, *BRAF* inhibitors were not approved by the United States Food and Drug Administration and thus neither *BRAF* testing nor *BRAF* inhibitor therapy represented a standard of care. *BRAF* inhibitors by present standards are typically utilized in melanoma patients who are symptomatic due to high tumor burden, rather than in patients who achieve “no evidence of disease” status after surgical resection of an isolated metastasis.

Molecular profiling of tumors is becoming a standard of care in breast, colon, and lung cancers as well as in melanoma. Cancer care is now largely shifting towards a personalized medicine approach. This case demonstrates the importance of somatic and germline analyses that can be a source of guidance in a personalized understanding of patient prognosis and tumor biology.

## Consent

Written informed consent was obtained from the patient for study of his tissue and for publication of patient-related data in accordance with the New York University Interdisciplinary Melanoma Cooperative Group protocol for patient enrollment and informed consent, which is approved by the NYU IRB. A copy of the consent is available for review.

## Abbreviations

AKT: v-akt murine thymoma viral oncogene homolog
BRAF: v-Raf murine sarcoma viral oncogene homolog B
CNV: copy number variation
CT: computed tomography
DNA: deoxyribonucleic acid
FFPE: formalin-fixed paraffin embedded
HMB-45: human melanoma black 45
IFNα: interferon-alfa
IL10: interleukin 10
IRB: Institutional review board
MBM: melanoma brain metastases
MIB1: mindbomb E3 ubiquitin protein ligase 1
MRI: magnetic resonance imaging
NCCN: National Comprehensive Cancer Network
NRAS: neuroblastoma RAS viral (v-Ras) oncogene homolog
NYU: New York University
OS: overall survival
PI3K: phosphatidylinositol 3-kinase
RFS: recurrence-free survival
SNP: single nucleotide polymorphism
TERT: telomerase reverse transcriptase
TIL: tumor infiltrating lymphocyte
WT: wild type
